# Cognitive Abilities and School Achievement: Addressing Challenges Across Adolescence

**DOI:** 10.3390/jintelligence13020021

**Published:** 2025-02-14

**Authors:** Gina C. Lemos, M. Adelina Guisande, Leandro S. Almeida

**Affiliations:** 1 Instituto Politécnico de Setúbal, Escola Superior de Educação, 2910-761 Setúbal, Portugal; 2 CIEd—Research Centre on Education, Institute of Education, University of Minho, 4710-057 Braga, Portugal; 3 Department of Developmental and Educational Psychology, Faculty of Psychology, University of Santiago de Compostela, 15782 Santiago de Compostela, Spain; mariaadelina.guisande@usc.es; 4 CIPsi—Psychology Research Center, School of Psychology, University of Minho, 4710-057 Braga, Portugal; leandro@psi.uminho.pt

**Keywords:** intelligence, academic achievement, adolescence, intelligence tests

## Abstract

Background: The school curriculum is increasingly aligned with real-world contexts and transversal skills. Simultaneously, conceptions of intelligence now emphasize contextual, motivational, and emotional dimensions. These shifts raise questions about the relevance of classical intelligence tests in predicting academic achievement, particularly during adolescence, a time of major curricular and developmental changes. Methods: Two independent samples of students, sixth–ninth grades (*n* = 1708) and tenth–twelfth grades (*n* = 3007), were randomly selected from public schools across Portugal. Cognitive abilities were measured by “Bateria de Aptidões Cognitivas” (BAC-AB), with nine subtests combining three contents (spatial, verbal, numerical) and three cognitive processes (comprehension, reasoning, problem solving). School achievement considers students’ grades in Portuguese and Mathematics. Results: Subtest scores were higher in advanced grades, particularly in early adolescence. The correlations between cognitive subtests and academic achievement suggested that alignment between test item content and curriculum subjects is more influential than cognitive processes. Subtest scores explained a larger proportion of variance in academic achievement in basic education than in secondary education. Conclusions: Curricular changes may reduce reliance on classical cognitive abilities for academic achievement, though correlations remain significant. More integrated cognitive skills are emphasized as the curriculum aims to prepare students for understanding complexity, adapting to societal changes, and applying knowledge to respond effectively to challenges in and outside of school.

## 1. Introduction

Thinking is a purposeful and active cognitive process through which individuals analyze, evaluate, and synthesize information to make sense of their surroundings (Roso 2019). It is not a passive or automatic activity but rather a deliberate process that requires intentionality, controlled engagement, and conscious effort to develop higher-order cognitive skills (Dere et al. 2021). In addition to setting humans apart from artificial systems, these cognitive processes provide the framework for handling difficult problems in a rapidly changing technological environment (Suomala and Kauttonen 2022). Recent advancements in Artificial Intelligence (AI) and Machine Learning (ML) have sparked debates regarding the uniqueness of human thinking, emphasizing its integration of logic, creativity, and emotional depth and adaptability (e.g., Cropley et al. 2022; Zhou and Lee 2024). Unlike AI, which excels in predefined algorithms and linear processing, human cognition adapts flexibly to dynamic environments, blending logic with intuition to define and solve complex problems and make decisions under uncertainty (Mogi 2024). Moreover, curiosity, self-guided inquiry, and reflective doubt are central to human cognition, encompassing flexible, intentional, and conscious cognitive processes that drive exploratory behaviors, knowledge acquisition, the pursuit of novel solutions, and the development of a sense of self (Sun et al. 2022; Suomala and Kauttonen 2022).

The capacity for conscious thinking is crucial in developing logical reasoning, informed decision-making, and addressing complex problems, particularly in a rapidly changing global context. The UNESCO report *Reimagining Our Futures Together* emphasizes the importance of education in cultivating deliberate, reflective cognitive processes that prepare individuals to navigate uncertainties and engage with global challenges (UNESCO 2021). Similarly, the OECD’s *Future of Education and Skills 2030* advocates for fostering cognitive skills essential for adaptation to technological, environmental, and societal changes (OECD 2019). The *European Commission’s Key Competences for Lifelong Learning* underscores reflective thinking as key to adaptability and personal growth (European Commission 2018). Roso (2019), in the report *‘Teaching thinking’ and ‘thinking to learn’: An urgent curriculum and pedagogical design challenge*, further highlights the need for curricula that prioritize structured thinking to enhance students’ capacity for lifelong learning and adaptability. These reports underscore the critical role of key competences in shaping future success by fostering adaptability, informed decision-making, and problem-solving skills. In fact, political decision-makers have been reshaping educational policies and school curricula to ensure that children and adolescents have the necessary skills for quality learning and full citizenship for individual, collective, and public good (Hughes 2020; OECD 2024) since “the future for our learners is not only unpredictable but, perhaps, unimaginable” (Roso 2019, p. 17). In Portugal, the country of focus for the current paper, the *Perfil dos Alunos à Saída da Escolaridade Obrigatória* (Martins et al. 2017) establishes a pivotal shared vision of education that guides the development of all students, irrespective of their individual school trajectories. Foundational competencies such as critical thinking, creative thinking, problem-solving, and analytical reasoning are presented as vital for understanding complexity, adapting to societal changes, and applying knowledge effectively to real-world situations (e.g., Guamanga et al. 2024; Halpern and Dunn 2021; Park et al. 2021; Saiz and Rivas 2023; Thornhill-Miller et al. 2023; Yu and Zin 2023). These abilities are systematically embedded across the profile’s eight competence areas, ensuring their interdisciplinary development and practical relevance. For example, reasoning and problem-solving are emphasized as essential tools for addressing real-world challenges, while scientific and technological literacy enables students to interpret and act upon natural and social phenomena. The framework also prioritizes metacognitive skills, such as self-regulation and reflective thinking, which empower students to internalize knowledge and transfer it across various contexts, fostering adaptability and lifelong learning. Additionally, by situating cognitive skills within a broader value system—promoting sustainability, equity, and democratic citizenship—the profile ensures that these abilities transcend academic boundaries. They are pivotal for scholastic success and for practical utility in fostering active citizenship, resilience, and ethical decision-making in complex and unpredictable environments.

Central to learning and adaptive functioning across various domains of life, including academic achievement and problem-solving in real-world contexts, is *g* or general intelligence (Deary et al. 2007; Sternberg 2019). The Cattell–Horn–Carroll (CHC; Schneider and McGrew 2018) theory of intelligence provides a comprehensive hierarchical framework for understanding human cognitive abilities, organized into three strata. At the highest level, Stratum III encompasses *g*, a general cognitive ability that influences performance across a wide range of cognitive tasks, reflecting the shared variance among them. Stratum II identifies eight to ten broad abilities: fluid intelligence (Gf) refers to the cognitive ability to reason, solve novel problems, and think abstractly, independent of previously acquired knowledge or experiences, i.e., related to potential; crystallized intelligence (Gc) reflects the accumulation of knowledge, information, and skills acquired through experience, education, and acculturation, i.e., related to the execution of potential; quantitative knowledge (Gq) pertains to the acquired store of declarative and procedural mathematical knowledge; visual–spatial abilities (Gv) relate to the manipulation and interpretation of visual shapes, forms, or images; auditory processing (Ga) refers to the ability to analyze and synthesize auditory information; kinesthetic abilities (Gk) reflect the ability to use one’s body effectively for expressive and goal-directed purposes; short-term memory (Gsm) captures the ability to hold and process information temporarily, while long-term storage and retrieval (Glr) involves the effective retention and recall of information over time; processing speed (Gs) reflects the efficiency of cognitive operations; decision/reaction time (Gt) pertains to response times to stimuli. Stratum I is composed of specific abilities, providing a more detailed perspective of cognitive functioning. Cognitive assessments, including standardized general intelligence tests, are very often considered as one of the most privileged sources of information to diagnose learning disabilities or to infer about subsequent school success (Benson et al. 2019; Saklofske et al. 2013). Indeed, a broad measure of intelligence is a well-established predictor of school achievement (Sternberg 2019; Zaboski et al. 2018). Deary et al. (2007) conducted a 5-year prospective longitudinal study of over 70,000 children to investigate the relationship between psychometric intelligence at age 11 and educational achievement in national examinations across 25 academic subjects at age 16. The findings demonstrated a strong association, with a latent intelligence trait (Spearman’s g) showing a correlation of 0.81 with a latent trait of educational achievement (GCSE scores). General intelligence emerged as a significant predictor of success across all 25 subjects, accounting for 58.6% of the variance in Mathematics and 48% in Language (English). Roth et al.’s (2015) extensive psychometric meta-analysis on the relation between intelligence and school grades, involving 240 independent samples with 105,185 participants, clearly showed that intelligence is a very strong predictor of school grades, school-related factors (i.e., school subjects and grade level) affect the relationship, and intelligence has its highest population correlation of 0.54 in subject domains which focus on content.

More recently, Lozano-Blasco et al. (2022) conducted a meta-analysis of 27 studies, encompassing a total sample of 42,061 participants, to examine in-depth the relationship between types of intelligence and academic performance across different educational levels. This study examined how different aspects of intelligence influence academic performance. Among the seven models analyzed, the country of residence emerged as the strongest predictor, explaining 45% of the variance, followed by the type of intelligence model, which accounted for 35%. The findings emphasize the significance of general and implicit intelligence in academic success, with implicit intelligence (i.e., one’s belief in their own abilities) showing a stronger association with academic achievement than general intelligence. Moreover, Brandt et al. (2020) examined the associations between personality traits, cognitive ability, and academic performance in a sample of 12,915 ninth-grade students. They investigated whether the relationships between the Big Five personality traits, fluid intelligence (including abilities such as working memory, reasoning, and processing speed), and academic performance (measured through grades and test scores) varied across subjects, specifically Language (German) and Mathematics. Cognitive ability explained residual variance in Language grades, with *R*^2^ contributions near 0% for Language and approximately 11% for Mathematics. For test scores, cognitive ability explained more variance in Mathematics (22%) than in Language (12%). Personality traits accounted for similar variance in both subjects, with slightly more variance explained in grades (*R*^2^ = 0.18 for Language; *R*^2^ = 0.15 for Mathematics) than in test scores (*R*^2^ = 0.04 for Language; *R*^2^ = 0.06 for Mathematics). Overall, cognitive ability had a stronger influence on test scores, while personality traits played a greater role in grades. Despite these subject-specific differences, the total variance explained by all five personality traits did not differ significantly between Language and Mathematics. Quílez-Robres et al. (2021) carried out a meta-analysis to investigate the relationship between academic achievement and motivational (motivation, self-concept, and self-esteem), emotional (emotional intelligence, emotional competence, and emotional well-being), and social factors (social intelligence, social competence, and social skills) in 15,777 children aged 6–12 years. The results revealed a moderate positive effect size for motivational factors (0.32) and social factors (0.21), and a small positive effect size for emotional factors (0.17). Moderating effects were identified, with age accounting for 65% of the variance in social factors and geographical area explaining 52% of motivational factors, 17% of emotional factors, and 76% of social factors. These findings highlight the importance of motivational and social factors in academic achievement, with age and geographical area serving as significant moderators. Moreover, Demetriou et al. (2023) conducted a review of research examining the relationships between cognitive processes, self-awareness, language, personality, and school performance. They proposed a comprehensive model illustrating how these factors interact across different developmental stages. The study emphasizes that general intelligence (g) forms the foundation for key mental processes, including executive functions, reasoning, language, self-awareness, and personality. The influence of g evolves with age, initially supporting executive and attentional processes in early childhood and later facilitating reasoning and self-awareness in adolescence and beyond. Additionally, personality traits tend to stabilize and exert greater influence during the adolescent years. The review identified three key findings regarding the predictors of school performance. First, when examined individually, cognitive abilities, self-awareness, language, and personality each account for approximately 20% of the variance in school performance. Second, when considered together, cognitive processes emerge as the strongest predictors, explaining over 50% of the variance, while self-concepts and personality traits contribute only 3–5%. In preschool, attention control and representational awareness play the most significant roles, whereas fluid intelligence becomes the dominant predictor in later years. Third, the factors influencing school performance evolve with age. In preschool, attention control and representational awareness account for roughly 85% of the variance. In primary school, a combination of fluid intelligence, language, and working memory explains 53%. By secondary school, predictors expand to include fluid intelligence, language, self-evaluation, and school-specific self-concepts, collectively accounting for approximately 70% of the variance, with personality traits gaining greater influence during adolescence. Further, Cortés Pascual et al. (2019) conducted a meta-analysis of 21 studies, encompassing a total of 7947 participants to investigate the relationship between executive functions and academic performance in primary education. The analysis revealed a moderately significant weighted effect size of *r* = 0.36, indicating that executive functions are a good predictor of academic performance in primary education. This effect was consistent across subjects, with similar results for Language (*r* = 0.35) and Mathematics (*r* = 0.36). The study also highlighted the importance of specific executive functions, such as working memory, in predicting academic success.

In summary, these studies suggest that the influence of cognitive ability on school achievement fluctuates over time. More specifically, the proportion of variance in school achievement attributed to cognitive abilities gradually declines throughout schooling, highlighting the need to incorporate motivational and personality variables in future research. Based on the reviewed literature, there are four key reasons that may explain this pattern.

The first line of force is adolescence as a critical developmental window for the transformation of fluid intelligence (Gf) into crystallized intelligence (Gc), as proposed by Cattell’s (1971) Investment Theory. During this period, Gf reaches its peak, enabling adolescents to process complex and abstract information with increased efficiency (Cattell 1971; Ganzach 2021; Lövdén et al. 2020). This cognitive flexibility allows them to engage with new learning experiences, gradually converting problem-solving abilities into accumulated knowledge and expertise (Scherrer et al. 2024). The extent of this transformation depends on both external and internal factors. Access to quality education, familial support, and diverse learning opportunities shape the development of Gc (Ganzach 2021). Likewise, intrinsic motivation, intellectual curiosity, and persistence play essential roles in guiding adolescents to actively invest their cognitive resources (Ackerman 2007; Alaybek et al. 2021; Duckworth et al. 2007; von Stumm and Ackerman 2014). As schooling progresses, the growing reliance on Gc explains the decreasing predictive power of general cognitive ability in academic performance (Lemos et al. 2014). While early education emphasizes basic cognitive skills closely linked to Gf, higher educational levels demand specialized knowledge, making domain-specific expertise increasingly relevant (Ackerman 1996; Horn and Noll 1997; Schneider and McGrew 2018).

The second line of force is the growing importance of non-cognitive, socio-emotional, and motivational factors in shaping students’ academic performance. Motivation, self-concept, and self-esteem show a moderate positive effect on school achievement (Quílez-Robres et al. 2021). In some cases, implicit intelligence—students’ belief in their own abilities—emerges as a strong predictor, with a greater impact on academic achievement than general intelligence (Lozano-Blasco et al. 2022). Personality traits further contribute to academic performance, particularly in grades, where their influence exceeds that of cognitive ability (Brandt et al. 2020). Despite subject-specific variations, personality traits account for a similar proportion of variance across disciplines. Self-awareness and self-evaluation also gain importance over time, contributing 3–5% to school performance, with personality traits stabilizing and exerting greater influence during adolescence (Demetriou et al. 2023). These findings highlight the substantial and enduring role of non-cognitive and motivational traits in academic achievement, particularly as students progress through their education.

The third line of force explores the growing complexity of curriculum content and the cognitive processes required at each stage of schooling. As students progress, the demands on their cognitive abilities change. In early education, attention control and representational awareness are crucial (Demetriou et al. 2023). In primary school, fluid intelligence, language, and working memory become more important. Fluid intelligence, which includes reasoning and problem-solving, is key as students face more complex and abstract content. By secondary school, the curriculum becomes even more specialized. As the curriculum’s complexity increases, executive functions like working memory also gain importance. Studies by Cortés Pascual et al. (2019) show that working memory is a significant predictor of academic performance, particularly in subjects like Mathematics and Language. Ultimately, the ability to apply cognitive resources effectively is vital as the curriculum becomes increasingly challenging.

The fourth line of force concerns the limitations of traditional intelligence tests in explaining the functions underlying human cognition and predicting academic achievement throughout schooling. While intelligence tests have long been used to measure cognitive abilities, they fail to account for the full range of cognitive processes involved in learning. These tests predominantly focus on content knowledge and do not sufficiently cover functions such as executive functions, which include working memory, attentional control, and cognitive flexibility (Diamond 2013). These executive functions play a crucial role in academic success, especially as the complexity of the curriculum increases throughout schooling. Intelligence tests also overlook more global cognitive functions, such as problem-solving, critical thinking, and metacognition—abilities that become increasingly important as students progress through their education (Sternberg 2019). Furthermore, research shows that intelligence, particularly general intelligence (g), is a strong predictor of academic performance in subjects that focus on content (Deary et al. 2007). However, this relationship diminishes as tasks require higher-order cognitive skills, such as executive control and self-regulated learning. As schooling advances, these skills become more essential for success, making traditional intelligence tests less effective at predicting performance in more complex academic contexts. Thus, while intelligence tests remain valuable for assessing certain cognitive abilities, they fall short in capturing the broad range of cognitive functions necessary for academic achievement across different stages of education.

Until recently, Portugal had an instrument for assessing intelligence designed specifically for the Portuguese population and aimed at adolescents, but it only measured a single cognitive process, reasoning or Gf, in different contents—“Bateria de Provas de Raciocínio” (BPR; Almeida and Lemos 2007). Therefore, there was a need to better characterize the cognitive abilities of Portuguese students across not only various domains or contents but also in different cognitive processes of increasing complexity, firmly grounded on the Cattell–Horn–Carroll (CHC) hierarchical model of intelligence which combines the most theoretical and empirical evidence regarding the structure of cognitive abilities and serves as a guide for the development and validation of the major intelligence tests used nowadays (Schneider and McGrew 2018)—“Bateria de Aptidões Cognitivas” was developed (BAC-AB; Lemos and Almeida 2015). BAC-AB is aimed at evaluating, in combination, three different domains or item contents (verbal, numeric, and spatial) considered as the three major contents that best represent intellectual human capacity, and three cognitive processes (comprehension, reasoning, and problem solving) at the pivotal development period of adolescence.

Studies with Portuguese representative samples of adolescents who completed these intelligence batteries (e.g., Lemos and Almeida 2019; Lemos et al. 2014; Soares et al. 2015) have consistently demonstrated: (i) a significant positive relationship between cognitive performance and academic achievement across all grades of schooling; (ii) a progressively weaker and heterogenous correlation between scores on cognitive subtests and school achievement in curricular subjects as students advance through successive school grades; (iii) the critical role of content alignment between cognitive subtests and curricular subjects in shaping these correlations, irrespective of grade level, with particularly strong associations observed between verbal cognitive subtests and Language school achievement, and between numerical cognitive subtests and Mathematics school achievement; and (iv) within the framework of differentiation of cognitive abilities in adolescence, there is a convergence of a more general ability or fluid intelligence along with more differentiated cognitive abilities depending, for instance, on school experiences, motivation, or interests.

The four primary aims of this study can be operationalized into the following research questions:

- What is the trend of students’ achievement in different cognitive subtests in grades sixth to ninth and tenth to twelfth? By focusing on variations in cognitive achievement across grades and these age groups, this study seeks to provide insights concerning changes in cognitive performance during adolescence.
- What is the relationship between cognitive achievement and school achievement throughout schooling? By adopting a cross-sectional approach, this study endeavors to examine how cognitive abilities relate to school achievement, particularly in subjects like Language (Portuguese) and Mathematics, at different stages of schooling and adolescence. This involves exploring and identifying any relevant correlation pattern across grades and within young and old adolescent samples.
- Which cognitive subtests can significantly predict school achievement of Portuguese and Mathematics in different grades, and what are the differences in their predictive power in different grades? By investigating the predictive relationship between school achievement in these subjects and the nine cognitive subtests, which assess three distinct cognitive processes across three different content domains, this study intends to provide insights into the unique contributions of these abilities to school achievement in each subject during this developmental period.
- To what extent do traditional intelligence tests remain relevant in explaining school achievement, given the evolving curricula and the increasing emphasis on transversal skills such as critical and creative thinking? This discussion seeks to explore how conventional intelligence assessments remain pertinent in light of the growing emphasis on broader, multifaceted skills in educational settings.

## 2. Materials and Methods

### 2.1. Participants

This study involved two independent samples of students attending sixth through ninth grades and students attending tenth to twelfth grades, from Portuguese public basic and secondary schools. The first sample comprised 1708 young adolescents (*mean age* = 12.88, *SD* = 1.27, ranging from 10 to 18 years, 49.6% male and 50.4% female), whereas the second sample included 3007 old adolescents (*mean age* = 16.52, *SD* = 1.24, ranging from 13 to 22 years, 45.8% male and 54.2% female).

The selection of schools met the criteria of randomness within the respective geographical area (NUT II) of all the regions of mainland Portugal (north, center, Lisbon and Tagus Valley, and south) and the autonomous regions of Madeira and the Azores, and the socio-cultural heterogeneity of the school population according to the school categorization of the Ministry of Education. Schools considered to be educational territories of priority intervention (i.e., with a predominantly underprivileged or vulnerable population) were excluded.

In order to ensure fairness in the analysis of the educational reality, the secondary school sample took into account the diversity of curricular options. Thus, the second sample consisted of students attending courses aimed at pursuing higher education degrees, scientific–humanistic courses (Level 3 qualifications on the National Qualifications Framework), including Science and Technology (n = 1378) and Socioeconomic Sciences and/or Languages and Humanities (n = 873).

### 2.2. Instruments

**Figure 1:**
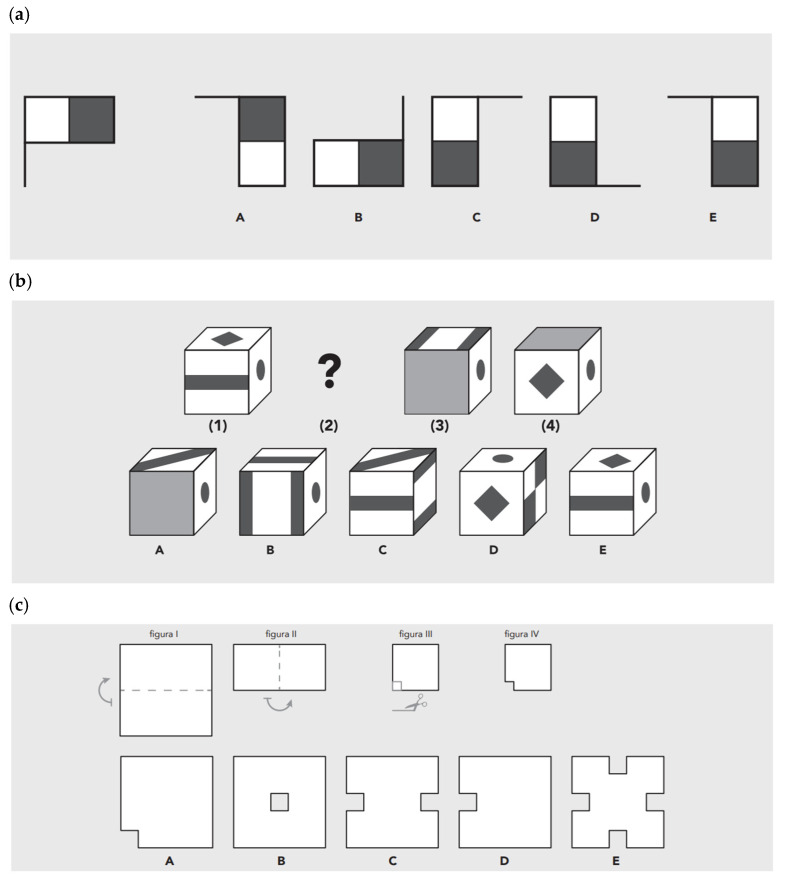

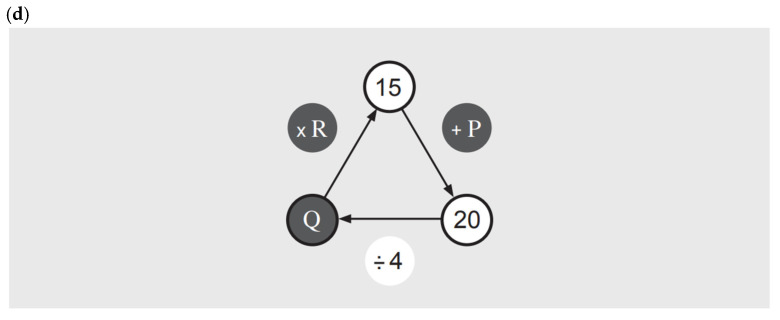
Example items from the administered intelligence battery. Figure Rotation’s example item (**a**). Cubes Sequences’ example item (**b**). Movements and Shapes’ example item (**c**). Calculus’ example item (**d**).

Cognitive abilities were measured by “Bateria de Aptidões Cognitivas” (BAC-AB; Lemos and Almeida 2015), which aims to assess, in combination, three domains or item contents considered to be the most representative of human intellectual capacity (spatial, verbal, numerical) in three cognitive processes of increasing complexity (comprehension, reasoning, problem solving). Thus, BAC-AB consists of nine time-limited subtests: Synonyms subtest (twenty-four items and four min of administration time; e.g., The group **scout**. A. Lookout B. Analyst C. Coordinator D. Broker E. Director), Analogies subtest (twenty-four verbal analogies and four min of administration time; e.g., **Foot** is to ___________ as **shoe** is to **glove**. A. Finger B. Ring C. Arm D. Sock E. Hand), Expressions subtest (twelve idiomatic and other expressions items and six min of administration time; e.g., **Rita gets up with the chickens.** A. Rita sleeps near a chicken coop. B. Rita hears the cackling of chickens. C. Rita is an early riser. D. Rita likes to sing when she wakes up. E. Rita usually wakes up early.), Figures Rotation (twenty mental rotation items and seven min of administration time; e.g., Figure 1a), Cubes Sequences (twenty spatial orientation and cube rotation series and ten min of administration time; e.g., Figure 1b), Movements and Shapes (twenty spatial and mechanical problem-solving items and twelve min of administration time; e.g., Figure 1c), Calculus (eight calculation items and ten min of administration time; e.g., Figure 1d), Numeric Sequences (fifteen numerical series and ten min of administration time; e.g., 2 1 3 1 **P** 1 5 **Q** 6), and Problems (twelve numerical problem-solving situations and fifteen min of administration time; e.g., There are 57 ladies and 39 men on a dance floor. Each of the men made a pair with a lady. The other ladies formed pairs with one another. (a) How many pairs of people were on the dance floor? (b) How many pairs were formed only by ladies?).

Young adolescents, from the first sample, performed the version designed for students from sixth to ninth grades (BAC-A), and old adolescents, from the second sample, performed the version designed for students from tenth to twelfth grades (BAC-B). BAC-AB has anchor items; more specifically, 50% of the items are common to both versions of the battery: the easiest items in the BAC-A were not included in the BAC-B, and the most difficult items in the BAC-B were not included in the BAC-A.

Previous studies show positive evidence of reliability and validity of BAC-AB’s subtests for both versions. The internal consistencies ranged from 0.70 (Analogies) and 0.88 (Calculus) for BAC-A and ranged from 0.82 (Expressions) and 0.93 (Calculus) for BAC-B. Criterion validity was estimated using Pearson’s correlation between cognitive performance and school achievement across various subjects. The results showed (1) a significant positive association between cognitive subtest performance and academic achievement in all seven school grades studied, and (2) the magnitude of the coefficients was higher and more consistent in the earlier school grades, progressively becoming lower and more variable from the ninth grade onward (Lemos and Almeida 2019). School achievement was assessed using students’ grades in Language (Portuguese) and Mathematics curricular subjects at the conclusion of the first term of the school year (equivalent to the first quarter). For students attending sixth through ninth grades (i.e., basic education), grades were reported on a scale from 1 to 5, with 3 representing the minimum approval score. For students attending tenth through twelfth grades (i.e., secondary education), grades were provided on a scale from 1 to 20, where a score of 10 was the minimum required for approval.

### 2.3. Procedures

Higher authorities, specifically the National Data Protection Commission, the Directorate General for Education of the Ministry of Education, and the Ethics Committee of the original higher education institution, granted prior authorization for all data collection procedures. Students were briefed of the study’s objectives, the voluntary nature of their participation, and the confidentiality of their responses before providing their informed assent after receiving letters of informed consent from their parents. The BAC-AB was administered by psychologists who had obtained special training for the purpose. The instructions for each subtest were strictly followed and included clear examples. Data collection was conducted during regular class hours with a maximum of 28 students per class, following the teachers’ consent. To reduce student fatigue, the nine subtests were administered over two separate days (90 min each day) within the same week or across consecutive weeks. The order of subtest administration was consistent and designed to account for the content and processes assessed, ensuring that no two subtests of the same type (verbal, numerical, or spatial) were administered consecutively. Students completed BAC-AB subtests in approximately 80 min.

Data on students’ school achievement in curricular subjects were officially provided by the schools’ administrative services, ensuring accuracy and consistency in the reporting process.

### 2.4. Data Analysis

The data were analyzed using IBM SPSS software, version 28.0. Since the skewness and kurtosis values for the distribution of results across subtests and curricular subjects over different school grades were minimal and consistently below 1.0, parametric statistics were chosen to analyze mean differences and correlations. The significance of differences among schooling groups on BAC-AB subtests was assessed using a one-way ANOVA, and the intergroups differences were examined using the *Scheffe* test (with a threshold of at least *p* < .05 required). For the linear regression analysis, the enter method was employed to identify which BAC-AB subtests could predict students’ school achievement in Portuguese and Mathematics.

## 3. Results

Table 1 shows the means and standard deviations of the results obtained by the students in the nine cognitive subtests and in school achievement, namely in Portuguese and Mathematics curricular subjects, by school grade (between the sixth and ninth school grades, i.e., the last four years of basic education).

Analyzing the means obtained, a certain variability in values is observed depending on the specificity of the subtest and the students’ school grade. Regarding school grade, the differences are more pronounced and generalized across the various subtests, with the sole exception occurring in the Calculus subtest. In this subtest, the mean score of sixth-grade students surpasses that of seventh-grade students, and the mean score of eighth-grade students is slightly higher than that of ninth-grade students. Thus, the observed differences indicate a progressive increase in mean scores on the cognitive subtests as students advance through school grades, with this difference being statistically significant: Synonyms subtest (*F*(3, 1656) = 98.460, *p* < .001, *η*^2^ = 0.165); Analogies subtest (*F*(3, 1630) = 38.738, *p* < .001, *η*^2^ = 0.070); Expressions subtest (*F*(3, 1662) = 147.257, *p* < .001, *η*^2^ = 0.242); Figures Rotation subtest (*F*(3, 1660) = 19.702, *p* < .001, *η*^2^ = 0.038); Cubes Sequences subtest (*F*(3, 1619) = 51.111, *p* < .001, *η*^2^ = 0.088); Movements and Shapes subtest (*F*(3, 1662) = 42.343, *p* < .001, *η*^2^ = 0.080); Calculus subtest (*F*(3, 1665) = 10.900, *p* < .001, *η*^2^ = 0.021); Numeric Sequences subtest (*F*(3, 1662) = 41.356, *p* < .001, *η*^2^ = 0.075); and Problems subtest (*F*(3, 1665) = 20.947, *p* < .001, *η*^2^ = 0.034).

Analyzing the differences in mean scores when comparing students across the four school grades (*Scheffe* test), two predominant patterns of differences are observed. In Synonyms and Expressions subtests, there is a statistically significant difference between all grades, with scores progressively favoring students in the subsequent school grade (sixth grade < seventh grade < eighth grade < ninth grade).

In contrast, for the subtests of Figure Rotation, Number Series, Movements and Shapes, Analogies, Cube Sequences, and Problems, statistically significant differences are observed when comparing students in the sixth and seventh grades with those in the eighth and ninth grades, but not between the latter two groups (sixth grade, seventh grade < eighth grade, ninth grade).

Finally, a different pattern emerges in the Calculus subtest, where only the mean score of seventh-grade students is significantly lower when compared to the eighth and ninth grades (seventh grade < eighth grade, ninth grade).

In summary, as expected, differences in mean scores across cognitive subtests are found when taking into account school grade, favoring students in higher grades. Some variations from this pattern require closer examination of the content of the items in certain subtests or the specific characteristics of school grades, which will be further addressed in the discussion section.

Regarding school achievement in Portuguese and Mathematics, with student grades ranging from 1 to 5, similar mean scores were observed in both subjects across the four school grades, with a slight advantage for Portuguese in the seventh and eighth grades, and for Mathematics in the ninth grade. On the other hand, considering the standard deviation, it is consistently higher in Mathematics, indicating greater variability in student grades in this subject. When analyzing the mean scores by school grade, statistically significant differences were found in both Portuguese (*F*(3, 1660) = 10.859, *p* < .001, *η*^2^ = 0.021) and Mathematics (*F*(3, 1661) = 17.022, *p* < .001, *η*^2^ = 0.032). The pattern of differentiation in mean scores across school grades with statistical significance (*Scheffe test*) follows the same trend in both subjects: sixth-grade students outperform those in the subsequent grades, with no significant differences between the seventh, eighth, and ninth grades (sixth grade > seventh, eighth, and ninth grades). In Mathematics, the difference is also significant when comparing students in the seventh and ninth grades, with ninth-grade students outperforming their seventh grade counterparts.

Table 2 presents the correlations between cognitive achievement in the nine subtests and school achievement in the two curriculum subjects, considering the students’ school grade.

Considering the magnitude of the correlation coefficients, it is clear that some cognitive subtests are more strongly associated with students’ school achievement than others. Specifically, the verbal and numerical content subtests show stronger associations with school achievement, while the figurative–spatial content subtests exhibit lower correlations, particularly the Figure Rotation and Movements and Shapes subtests. This pattern of correlations remains consistent across the school grades considered, with higher coefficients observed for sixth-grade students. Finally, while the three verbal content subtests (Synonyms, Analogies, and Expressions) show similar correlation coefficients for both Portuguese and Mathematics, the figurative–spatial and numerical content subtests are more strongly correlated with students’ school achievement in Mathematics, particularly the numerical content subtests.

Given the curricular and age-specific characteristics of students across the four school grades, the regression analysis was conducted separately for each grade. Proceeding to the regression analysis using the nine cognitive subtests and each of the curricular subjects by school grade, Table 3 presents a summary of the coefficients obtained (standardized beta, t, and statistical significance). The regression models were statistically significant for all four school grades and the two subjects considered: sixth grade—Portuguese: *R*^2^*Adj* = 0.30, *F*(9, 303) = 15.367, *p* < .001; Mathematics: *R*^2^*Adj* = 0.47, *F*(9, 303) = 30.604, *p* < .001; seventh grade—Portuguese: *R*^2^*Adj* = 0.22, *F*(9, 467) = 15.483, *p* < .001; Mathematics: *R*^2^*Adj* = 0.36, *F*(9, 467) = 29.615, *p* < .001; eighth grade—Portuguese: *R*^2^*Adj* = 0.32, *F*(9, 435) = 23.698, *p* < .001; Mathematics: *R*^2^*Adj* = 0.30, *F*(9, 435) = 21.999, *p* < .001; ninth grade—Portuguese: *R*^2^*Adj* = 0.30, *F*(9, 311) = 15.428, *p* < .001; Mathematics: *R*^2^*Adj* = 0.41, *F*(9, 311) = 25.413, *p* < .001. This dataset reveals that the cognitive subtests explain a higher percentage of variance in students’ grades in Mathematics compared to Portuguese, a pattern that does not hold for eighth grade. The explained variance is particularly notable in sixth and ninth grades, accounting for 47% and 41%, respectively.

As we can observe, grades in the Portuguese subject are primarily associated with subtests that have verbal content, with the Synonyms and Expressions subtests standing out. This is particularly evident for students in the eighth and ninth grades, and to some extent in the sixth grade. In the seventh grade, however, the Calculus subtest has a greater impact. It is also noteworthy that this numerical content subtest is associated with grades in Portuguese for students in the sixth, eighth, and ninth grades, albeit with lower coefficients. Lastly, the Cube Sequences subtest shows some impact on grades in Portuguese for students in the sixth and seventh grades.

Analyzing the impact of cognitive subtests on students’ grades in Mathematics, the Calculus subtest shows a higher impact across most grade levels. However, its effect is less pronounced for the eighth grade. The Synonyms subtest has a strong impact on sixth-grade students’ Mathematics grades. This significant effect is also observed in eighth-grade students, though to a lesser extent. To further explain Mathematics grades, other numerically oriented subtests play a role. The Problems subtest significantly impacts the sixth, eighth, and ninth grades, while the Number Sequences subtest contributes to the seventh and eighth grades. Finally, the Cube Sequences subtest explains Mathematics grades for the sixth, seventh, and eighth grades, as well as the Movements and Shapes subtest for seventh grade and the Expressions subtest for eighth grade.

The analysis of the results for secondary school students (tenth to twelfth grades) follows. Table 4 presents the mean and standard deviation of the results across the nine cognitive subtests by school grade, as well as in the two curricular subjects.

Some mean differences across school grades are observed in Synonyms and Expressions subtests, with higher scores in the upper grades. However, this pattern of differentiation in mean scores is not evident in certain subtests, such as Analogies and Numeric Sequences. When analyzing the statistical significance of the differences observed across school grades, differences were found for the following subtests: Synonyms (*F*(2, 2154) = 43.361, *p* < .001, *η*^2^ = 0.039), Expressions (*F*(2, 2151) = 52.318, *p* < .001, *η*^2^ = 0.046), Figures Rotation (*F*(2, 2151) = 3.093, *p* < .05, *η*^2^ = 0.003), Cubes Sequences (*F*(2, 2047) = 12.606, *p* < .001, *η*^2^ = 0.012), Movements and Shapes (*F*(2, 2151) = 28.979, *p* < .001, *η*^2^ = 0.026), Calculus (*F*(2, 2049) = 8.981, *p* < .001, *η*^2^ = 0.009), and Problems (*F*(2, 2028) = 18.592, *p* < .001, *η*^2^ = 0.018). No statistically significant differences were observed in Analogies (*F*(2, 2052) = 1.751, *p* = .173, *η*^2^ = 0.002) and Numeric Sequences (*F*(2, 2141) = 0.842, *p* = .431, *η*^2^ = 0.001) subtests. Based on pairwise comparisons between the three school grades (*Scheffe* test), the Synonyms, Expressions, and Problems subtests showed a sequential pattern of increasing scores across grades (tenth grade < eleventh grade < twelfth grade). For the Movements and Shapes and Cubes Sequences subtests, significant differences were found when comparing tenth and eleventh grades with the twelfth grade (tenth and eleventh grades < twelfth grade). In the Calculus subtest, statistically significant differences were observed between the tenth grade and the two subsequent grades (tenth grade < eleventh and twelfth grades). No statistically significant differences were found between the three grades for the Figure Rotation, Numeric Sequences, and Analogies subtests. Regarding school achievement, a progressive increase in mean scores is observed across grade levels in both Portuguese and Mathematics subjects: Portuguese (*F*(2, 2126) = 74.431, *p* < .001, *η*^2^ = 0.066) and Mathematics (*F*(2, 1865) = 46.899, *p* < .001, *η*^2^ = 0.048). Pairwise comparisons reveal that in both subjects, the mean scores increase significantly with each grade level (tenth grade < eleventh grade < twelfth grade).

Table 5 shows that the three verbal-content subtests, along with the three numerical-content subtests, exhibit higher correlations with school achievement in Portuguese and Mathematics. In contrast, the figurative–spatial subtests are less strongly correlated with achievement in these two curricular subjects, particularly the Figures Rotation and Movements and Shapes subtests. The Numeric Sequences subtest also shows lower correlations compared to the Calculus and Problems subtests, which are also numerical in content. This pattern of correlations remains consistent across the three grades analyzed. Lastly, the three verbal-content subtests present higher correlation coefficients with school achievement in Portuguese, whereas the other subtests do not show differentiation in their correlation coefficients when considering the curricular specifics of the two subjects analyzed.

Proceeding to the regression analysis using the nine cognitive subtests and each of the curricular subjects by school grade, Table 6 presents a summary of the coefficients obtained. The regression models were statistically significant for all three school grades and the two subjects considered: tenth grade—Portuguese: *R*^2^*Adj* = 0.263, *F*(9, 677) = 28.677, *p* < .001; Mathematics: *R*^2^*Adj* = 0.180, *F*(9, 627) = 16.771, *p* < .001; eleventh grade—Portuguese: *R*^2^*Adj* = 0.277, *F*(9, 730) = 32.504, *p* < .001; Mathematics: *R*^2^*Adj* = 0.171, *F*(9, 656) = 16.095, *p* < .001; twelfth grade—Portuguese: *R*^2^*Adj* = 0.278, *F*(9, 429) = 19.693, *p* < .001; Mathematics: *R*^2^*Adj* = 0.223, *F*(9, 357) = 12.299, *p* < .001. When considering the percentage of variance explained for each school grade, a lower percentage was observed in the Mathematics subject (18%, 17%, and 22% for the tenth, eleventh, and twelfth grades, respectively) compared to the Portuguese Language subject (26%, 28%, and 28% for the tenth, eleventh, and twelfth grades, respectively).

In all three school grades, the Synonyms subtest demonstrates the greatest impact on school achievement, both in Portuguese and Mathematics, with higher beta values observed in Portuguese. Other cognitive subtests are associated with school achievement in these subjects, but this association is statistically significant only for students in the tenth and eleventh grades. The sole exception in the twelfth grade is the Expressions subtest, which has a positive impact on school achievement in Portuguese. In the tenth and eleventh grades, both verbal subtests (Expressions and Analogies) and both numerical subtests (Calculus and Problems) show a positive impact on Portuguese school achievement, although in the eleventh grade, only the Calculus subtest shows statistical significance. In contrast, when analyzing results in Mathematics, fewer cognitive subtests show a significant impact, with the Calculus subtest being particularly noteworthy. The figurative content subtests, specifically Figure Rotation and Movements and Shapes, do not show relevance in explaining Portuguese and Mathematics achievement. In some cases, these subtests even show a negative association, with the exception of the Cubes Sequences subtest, which has a significant and positive impact on the Mathematics achievement for tenth-grade students.

## 4. Discussion

Intelligence, defined as the set of cognitive abilities, remains widely recognized in the literature as a strong predictor of students’ school achievement (Deary et al. 2007; Roth et al. 2015; Saklofske et al. 2013; Zaboski et al. 2018). However, school achievement is currently conceptualized as a multidimensional construct rather than just a measure of curricular learning (Martins et al. 2017; OECD 2019; Roso 2019; UNESCO 2021). Given this shift, intelligence measured by traditional tests may no longer be as relevant in explaining school achievement as it was in past decades (Fuchs et al. 2024).

Curricular objectives change qualitatively throughout schooling. At the same time, the structure of intelligence evolves from childhood to adolescence and then adulthood. These developmental changes may affect how strongly intellectual abilities influence school achievement (Cattell 1971; Demetriou et al. 2023; Heckman and Kautz 2012; Lemos and Almeida 2019; Sternberg 2019).

By analyzing the variations in cognitive achievement across grades and age groups (i.e., young and old adolescents), our first research question, the results reveal that performance in cognitive subtests is higher at more advanced grades. However, these improvements are not linear, nor do they exhibit the same magnitude across all cognitive subtests. This increase appears to be more consistent in subtests related to verbal content, which is among the most frequently engaged material in students’ daily school activities. The non-linearity and varying levels of improvement in cognitive achievement across subtests may be linked to differences in curriculum content and learning experiences at different educational levels. Roth et al. (2015) show that intelligence and academic performance are more closely related in subjects that rely heavily on verbal abilities. Similarly, the consistent improvement in verbal subtests in this study may reflect the frequent use of verbal content in daily school activities. In contrast, other cognitive skills may develop at different rates, depending on curricular demands and how explicitly they are reinforced in academic tasks. Thus, a successive improvement is observed between the sixth and ninth grades, with statistically significant results in the Synonyms and Expressions subtests. This reflects the cumulative benefits of linguistic exposure and a growing sophistication in language-related cognitive processes over time. The observed improvement in the Synonyms and Expressions subtests between the sixth and ninth grades can be associated with the developmental transition from fluid to crystallized intelligence during adolescence (Cattell 1971). As adolescents’ cognitive flexibility (Gf) peaks, they process complex language tasks more efficiently, converting abstract problem-solving abilities into accumulated linguistic knowledge (Gc) (Ganzach 2021; Lövdén et al. 2020). This process is fueled by increased linguistic exposure and engagement (Scherrer et al. 2024), leading to a growing sophistication in their language-related cognitive skills over time, which is evident in their enhanced performance on these subtests. However, this linear progression does not extend to the Analogies subtest. Although it involves verbal content, it focuses more on reasoning than on vocabulary or verbal comprehension. While Synonyms and Expressions are related to verbal comprehension and vocabulary, which involve both crystallized intelligence (Gc) and cognitive flexibility (Ganzach 2021; Lövdén et al. 2020), the Analogies subtest is more focused on abstract reasoning and pattern recognition, which are closely tied to fluid intelligence (Gf) (Ackerman 1996; Cattell 1971). This difference in cognitive demands may help explain why the Analogies subtest does not show the same linear progression seen in the verbal subtests, as it taps into reasoning abilities that do not necessarily develop in a uniform way during adolescence (Scherrer et al. 2024). While vocabulary-based tasks benefit from accumulated knowledge, reasoning tasks like analogies require flexible thinking that peaks earlier in adolescence and may not follow the same gradual growth (Lemos et al. 2014). The increase in means between the sixth and ninth grades is also not linear in the figurative–spatial and numerical content subtests. This is besides the fact that numerical content is similarly prominent in students’ daily academic activities. The non-linearity in the increase observed in Mathematics’ grades may be linked to the significant number of students experiencing learning difficulties and underachievement in this subject (Instituto de Avaliação Educativa 2017a, 2017b, 2024; OECD 2023). Students may disengage or distance themselves from their learning due to a lack of previous knowledge, low motivation, and diminished self-efficacy perceptions (Demetriou et al. 2023; Lozano-Blasco et al. 2022; Quílez-Robres et al. 2021). Moreover, the decline in seventh graders’ performance on the Calculus subtest can be attributed to the curriculum shift from arithmetic fluency to more abstract concepts, the growing reliance on technology reducing the need for manual calculations, and developmental changes in students’ cognitive abilities (Ministério da Educação 2018a, 2018b).

Analyzing the results of secondary education students (i.e., the tenth to twelfth grades), the Synonyms and Expressions subtests reveal a successive increase in means, with statistical significance, as students progress through the grades. This linear trend is also observed in the Problems subtest. Given that this subtest also involves verbal content in the formulation or presentation of the problems, and requires comprehension of these statements for their resolution, these results further emphasize the relevance of students’ vocabulary and verbal comprehension. Two interpretations can explain these results. First, the influence of schooling or the curriculum on the development of cognitive abilities, as observed in younger adolescents between the sixth and ninth grade. Second, the growing complexity of curriculum content. This complexity demands the development of more sophisticated cognitive abilities, such as problem solving with verbal content (Cortés Pascual et al. 2019; Demetriou et al. 2023; Ganzach 2021; Scherrer et al. 2024). In summary, the increase in subtest scores is more linear and pronounced from sixth to ninth grade compared to tenth to twelfth grade, which aligns with the authors’ proposition that fluid intelligence (Gf) progressively stabilizes as individuals approach the end of adolescence (Lövdén et al. 2020; Scherrer et al. 2024). Simultaneously, the increase in cognitive performance with respect to grade and age is more pronounced in subtests involving verbal content and, though less distinctly, numerical content. This suggests the impact of curriculum and formal schooling on the differentiation of cognitive abilities during adolescence, as posited by the Cattell’s Investment Theory (Cattell 1971).

Before addressing our second research question, it is essential to acknowledge the decline of students’ school achievement in both foundational subjects (Portuguese and Mathematics) during the transition from the sixth grade to the seventh, eighth, and ninth grades. In contrast, a successive increase in school achievement in Portuguese and Mathematics is observed as students progress from tenth grade to eleventh grade and from eleventh grade to twelfth grade. This divergent pattern may be attributed to several factors, including the onset of adolescence and the socioemotional challenges particularly affecting students in the seventh to ninth grades (i.e., ages 13 to 15), making academic engagement a more complex and demanding process for these students. In turn, as access to higher education in Portugal is governed by a *numerus clausus* system, where admission to courses and institutions is based on the ranking of candidates according to access scores that combine school achievement in secondary education and national entrance exams, students may increasingly engage in their curricular learning as they progress through secondary education, setting the goal of higher education admission as a key component of their vocational or career aspirations.

Focusing on our second research question, and examining the relationship between cognitive subtests and students’ school achievement from the sixth to ninth grades, the results show higher correlations for subtests involving verbal and numerical content, which are more closely associated with the two curriculum subjects considered in this study. In contrast, subtests involving figurative–spatial content, particularly Figure Rotation and Movement and Shapes subtests, show weak correlations with school achievement. This correlation pattern is also observed among students in the tenth to twelfth grades, further highlighting the relevance of the subtest content for the magnitude of the correlation coefficients found separately to Portuguese and Mathematics. Again, the Figure Rotation and Movement and Shapes subtests show lower correlations with school achievement for students in the tenth to twelfth grades, potentially reflecting the lesser relevance of figurative–spatial content in explaining school achievement, particularly when not associated with reasoning tasks (e.g., Cubes Sequences subtest). Additionally, verbal subtests are more strongly associated with school achievement in both Portuguese and Mathematics for students in the tenth to twelfth grades, suggesting their broader applicability across various learning and performance contexts, while numerical subtests are more strongly correlated only with achievement in Mathematics (Lemos and Almeida 2019; Lemos et al. 2014; Soares et al. 2015). On the other hand, the low correlations between school achievement and the Numeric Sequences and Analogies subtests suggest that reasoning processes (inference and application of relations) become less decisive for learning and school achievement as students progress through adolescence and approach the end of compulsory education. Again, rather than cognitive processes considered in isolation, skills based on mastery of content closely related to the specific curriculum subjects appear to be more relevant for school achievement (Diamond 2013; Lemos et al. 2014; Roth et al. 2015; Soares et al. 2015).

To address our third research question—identifying which cognitive abilities best predict school achievement in Language (Portuguese) and Mathematics subjects during adolescence—a regression analysis was performed, with school achievement as the criterion variable and the nine cognitive subtests as predictor variables. The results of the regression analysis suggest a progressive decrease in the percentage of variance in school achievement explained by the cognitive subtests as we move from young adolescents (sixth to ninth grades) to old adolescents (tenth to twelfth grades). This decrease in values occurs in Portuguese and in Mathematics, even more expressive in Mathematics. These results suggest a diminishing impact of the cognitive variables assessed through these subtests on explaining students’ school achievement as they progress through their education and adolescence. This may indicate the growing relevance of other psychological and educational variables in shaping school achievement (Ackerman 2007; Alaybek et al. 2021; Brandt et al. 2020; Duckworth et al. 2007; Ganzach 2021; Ganzach and Zisman 2022; Heckman and Kautz 2012; Quílez-Robres et al. 2021). Lastly, Synonyms and Calculus are the subtests more relevant and frequent to explain the variance of academic achievement in Portuguese and Mathematics from the sixth to ninth grades. In these four grades, Expressions also tend to have a contribution to explain achievement in Portuguese, and Numeric Sequences and Problems to explain achievement in Mathematics. Considering students from the tenth to twelfth grades, the Synonyms subtest emerges as the most influential and consistent factor affecting school achievement in both Portuguese and Mathematics across the three grades, with the Calculus subtest being somewhat relevant to school achievement in Mathematics and the Expressions subtests influencing school achievement in Portuguese. These findings indicate a significant influence of cognitive abilities on school achievement, particularly when these abilities are reflected in tasks involving verbal and numerical content. Once again, this highlights the interaction between cognitive abilities and formal schooling during adolescence, consistent with the dynamic relationship between instructional content and cognitive achievement and the theory of progressive cognitive differentiation (Cattell 1971; von Stumm and Ackerman 2014). In an attempt to explain the diminishing impact of cognitive variables, the results of the regression analysis indicate a progressive decrease in the influence of cognitive abilities on school achievement from young adolescents (sixth to ninth grades) to old adolescents (tenth to twelfth grades). This trend aligns with the second line of force, which emphasizes the growing importance of non-cognitive, socio-emotional, motivational factors, and personality traits in shaping academic performance. In our findings, the cognitive subtests, particularly Synonyms and Calculus, play a significant role in explaining academic achievement in both Portuguese and Mathematics for young adolescents. However, for old adolescents, the relevance of these cognitive abilities diminishes, especially in Mathematics. This suggests that, over time, other non-cognitive factors, such as motivation, self-concept, and self-esteem (Quílez-Robres et al. 2021), begin to exert more influence on students’ academic success. For old adolescents, the Synonyms subtest remains the most consistent predictor of school achievement in both subjects, while the Calculus subtest continues to show some relevance in Mathematics. Non-cognitive factors, such as personality traits and self-awareness, likely play an increasingly important role during this period. Studies show that personality traits significantly contribute to academic performance (Brandt et al. 2020), and self-awareness and implicit intelligence—students’ belief in their own abilities—become more relevant as students age (Demetriou et al. 2023; Lozano-Blasco et al. 2022). This shift from cognitive to non-cognitive factors aligns with the idea that as students mature, their academic performance is increasingly shaped by socio-emotional factors and personality traits, reflecting the dynamic relationship between cognitive abilities and factors like motivation and personality.

The fourth and final research question of this study examines the findings to analyze and discuss the relevance of classical psychometric measures of intelligence in explaining school achievement. This is particularly important within the context of a modern curriculum that increasingly emphasizes transversal skills. These skills, such as critical thinking, creative thinking, and problem-solving, are more closely related to complex and global cognitive abilities (e.g., Guamanga et al. 2024; Halpern and Dunn 2021; Park et al. 2021; Saiz and Rivas 2023; Thornhill-Miller et al. 2023; Yu and Zin 2023). Given the higher correlation coefficients observed in studies conducted a few decades ago (Deary et al. 2007; Roth et al. 2015; Saklofske et al. 2013; Zaboski et al. 2018), it is possible that school achievement today, particularly during adolescence, may be less strongly associated with traditional cognitive tests, often rooted in the classic intelligence test format. This shift could reflect a closer alignment of the current school curriculum with integrative cognitive abilities, while school achievement itself may now be more closely linked to affective and motivational factors, and less dependent on cognitive abilities alone (Ackerman 2007; Alaybek et al. 2021; Duckworth et al. 2007; Ganzach 2021; Ganzach and Zisman 2022; Quílez-Robres et al. 2021; von Stumm and Ackerman 2014). Another possible reason for the decrease in the explanatory capacity of academic achievement by traditional intelligence tests is the lack of sufficient coverage of the descriptive functions or abilities of cognitive functioning. Based on neuroscience, more attention is paid to executive functions, namely working memory, attentional control, and cognitive flexibility (Cortés Pascual et al. 2019; Diamond 2013), which are not sufficiently represented in classic intelligence tests. Similarly, intelligence defined as an adaptation capacity to contexts—for example, recognizing and defining a problem, mentally representing the problem and a strategy to solve, and monitoring the strategy’s effectiveness during and after problem solution (Sternberg 2019)—involves cognitive functions that are not so present in classical intelligence tests. Furthermore, this discrepancy may be accentuated when those cognitive functions appear more valued today in the objectives of the school curriculum, particularly the development of metacognition, problem solving, critical thinking, or self-regulated learning as school progresses. This evolution suggests that psychologists and educators should consider the potential relevance of more global forms of thinking in explaining the cognitive processes involved in learning and academic success.

The present study has several limitations that should be acknowledged and addressed in future research. The first limitation pertains to the study’s design. While this study provides comprehensive analyses of cognitive performance across two groups of adolescents (i.e., younger and older adolescents) at various educational stages (sixth, seventh, eighth, and ninth grades of basic education; and tenth, eleventh, and twelfth grades, of secondary education), along with its relationship to school achievement in Portuguese and Mathematics, the interpretations drawn must be approached with caution due to the cross-sectional nature of the design. This limitation may lead to potential deviations in the interpretation of the relationship between cognitive ability and academic achievement. In this regard, a longitudinal study would enable the examination of developmental changes, offering a deeper understanding of the evolution of cognitive and academic achievement and their interrelationship over time. This approach would contribute to providing more accurate guidance for future research, ultimately yielding more robust and detailed conclusions about the underlying mechanisms of the relationship between these variables. Further, although the BAC-AB has expanded the range of cognitive processes assessed through an instrument developed and validated for the Portuguese population, covering the three content areas or domains considered most representative of human intellectual ability, the study would benefit from an increased number of tests aimed at assessing latent factors; for example, second-order factors that appear proposed in CHC theory. In addition, future research could explore non-cognitive variables (e.g., motivation, need for cognition, persistence) and higher-order cognitive and metacognitive variables (e.g., critical thinking, creative thinking). Incorporating these variables into the research design would enable a more detailed and nuanced understanding of the mechanisms underlying the observed trends. Addressing this limitation in future studies will allow for a more comprehensive analysis of the factors influencing academic achievement during adolescence, as well as their impact on the various challenging real-life contexts.

Moreover, the measure of school achievement in the present study relies on teacher-assigned grades, which can be influenced by several factors, such as teacher biases, subjective interpretations of student performance, and varying grading practices depending on teachers, classes, and schools. These factors can lead to inconsistencies in the evaluation of school achievement. In other words, teacher-assigned grades may not always align with students’ actual performance on standardized tests. The availability of standardized national school achievement measures, such as those derived from national exams, would undoubtedly enhance the reliability of academic performance measures. It is important to clarify that in the Portuguese education system, national exams are only conducted at the ninth and twelfth grades. Unfortunately, we did not have access to the national exam results for students in these grades during the course of our study. Future research could benefit from using standardized national school achievement scores (at least in the grades in which they take place) which would enhance the reliability and generalizability of the results.

The practical implications of this study are crucial for psychologists in educational settings. By analyzing the predictive power of individual cognitive subtests, psychologists gain a clearer understanding of how abilities like verbal comprehension and mathematical calculation relate to academic achievement. Psychologists can use these insights to select subtests that are relevant to specific academic areas, ensuring more targeted assessments and interventions. In collaboration with teachers, psychologists can identify cognitive strengths and challenges, guiding the development of personalized teaching strategies and better address students’ individual needs. From this perspective, our analysis of young and old adolescents’ cognitive performance and school achievement throughout schooling underscores the need to further explore complex cognitive abilities for a more nuanced understanding of the cognitive processes that underpin academic success. 

## Figures and Tables

**Table 1:** Means, standard deviations (SD) in cognitive subtests and school achievement in Portuguese and Mathematics curricular subjects by school grade (from sixth to ninth school grades).

	Grades			
Subtests	Sixth Grade	Seventh Grade	Eighth Grade	Ninth Grade
Synonyms	7.05 (2.97)	8.44 (3.50)	10.20 (3.84)	11.27 (3.86)
Analogies	9.68 (3.35)	9.79 (3.50)	11.39 (3.86)	11.95 (3.50)
Expressions	10.34 (4.04)	11.97 (4.45)	14.51 (4.91)	16.77 (3.95)
Figures Rotation	9.19 (3.79)	9.47 (3.77)	10.60 (3.88)	11.00 (3.86)
Cubes Sequences	8.71 (4.13)	8.80 (4.27)	11.19 (4.48)	11.67 (4.24)
Movements and Shapes	8.78 (3.09)	9.36 (3.00)	10.61 (3.48)	11.15 (3.36)
Calculus	11.53 (4.91)	10.82 (5.75)	12.64 (6.44)	12.55 (6.11)
Numeric Sequences	10.46 (4.98)	11.29 (4.66)	13.38 (5.17)	13.82 (4.88)
Problems	7.82 (4.07)	8.02 (5.31)	9.82 (5.96)	10.69 (4.94)
**School achievement**				
Portuguese	3.33 (0.74)	2.99 (0.73)	3.08 (0.77)	3.06 (0.74)
Mathematics	3.35 (0.94)	2.90 (0.89)	2.99 (0.94)	3.09 (0.99)

**Table 2:** Correlation coefficients between cognitive subtests and academic achievement in Portuguese and Mathematics by school grade (from sixth to ninth grade).

Subtests	Sixth Grade		Seventh Grade		Eighth Grade		Ninth Grade	
	Port	Math	Port	Math	Port	Math	Port	Math
Synonyms	0.43 ***	0.40 ***	0.34 ***	0.35 ***	0.44 ***	0.32 ***	0.42 ***	0.34 ***
Analogies	0.46 ***	0.44 ***	0.35 ***	0.41 ***	0.27 ***	0.34 ***	0.34 ***	0.38 ***
Expressions	0.50 ***	0.37 ***	0.40 ***	0.37 ***	0.43 ***	0.35 ***	0.42 ***	0.36 ***
Figures Rotation	0.14 **	0.29 ***	0.11 *	0.21 ***	0.19 ***	0.28 ***	0.15 **	0.31 ***
Cubes Sequences	0.42 ***	0.50 ***	0.27 ***	0.36 ***	0.28 ***	0.38 ***	0.25 ***	0.44 ***
Movements and Shapes	0.38 ***	0.40 ***	0.24 ***	0.32 ***	0.22 ***	0.32 ***	0.11 *	0.29 ***
Calculus	0.46 ***	0.60 ***	0.43 ***	0.58 ***	0.28 ***	0.37 ***	0.34 ***	0.58 ***
Numeric Sequences	0.33 ***	0.49 ***	0.27 ***	0.40 ***	0.31 ***	0.44 ***	0.23 ***	0.45 ***
Problems	0.57 ***	0.64 ***	0.41 ***	0.52 ***	0.32 ***	0.40 ***	0.36 ***	0.58 ***

* *p* < .05, ** *p* < .01, *** *p* < .001 (two-tailed test). *Note*. Port, Portuguese school achievement; Math, Mathematics school achievement.

**Table 3:** Summary of the regression analysis coefficients by school grade (sixth to ninth grade) and school curricular subjects.

		Portuguese			Mathematics		
		β	*t*	*p*	β	*t*	*p*
**Sixth grade**	Synonyms	**0.194**	**3.333**	**<.001**	**0.205**	**4.048**	**<.001**
	Figures Rotation	−0.083	−1.578	.116	0.071	1.535	.126
	Numeric Sequences	−0.100	−1.683	.094	−0.012	−0.225	.822
	Expressions	0.177	2.879	.004	0.037	0.686	.494
	Movements and Shapes	0.012	0.203	.839	0.011	0.215	.830
	Analogies	0.102	1.640	.102	0.042	0.774	.440
	Calculus	0.205	2.908	.004	**0.312**	**5.063**	**<.001**
	Cubes Sequences	0.136	2.120	.035	0.169	3.015	.003
	Problems	0.065	0.848	.397	0.155	2.327	.021
**Seventh grade**	Synonyms	0.105	2.033	.043	0.029	0.609	.543
	Figures Rotation	−0.024	−0.508	.611	0.032	0.767	.444
	Numeric Sequences	0.071	1.461	.145	0.105	2.362	.019
	Expressions	0.089	1.613	.107	0.079	1.583	.114
	Movements and Shapes	0.009	0.183	.855	0.096	2.150	.032
	Analogies	0.094	1.765	.078	0.048	0.998	.319
	Calculus	**0.244**	**3.805**	**<.001**	**0.309**	**5.289**	**<.001**
	Cubes Sequences	0.113	2.262	.024	0.090	1.991	.047
	Problems	−0.054	−0.856	.392	0.064	1.119	.264
**Eighth grade**	Synonyms	**0.337**	**6.831**	**<.001**	0.114	2.284	.023
	Figures Rotation	0.002	0.046	.963	−0.006	−0.118	.906
	Numeric Sequences	0.088	1.793	.074	0.113	2.265	.024
	Expressions	**0.213**	**4.219**	**<.001**	0.101	1.989	.047
	Movements and Shapes	0.075	1.521	.129	0.094	1.875	.061
	Analogies	−0.011	−0.200	.841	−0.030	−0.555	.579
	Calculus	0.131	2.075	.039	0.180	2.813	.005
	Cubes Sequences	0.002	0.033	.974	0.110	1.971	.049
	Problems	−0.056	−0.807	.420	0.139	1.972	.049
**Ninth grade**	Synonyms	**0.246**	**4.108**	**<.001**	0.082	1.511	.132
	Figures Rotation	−0.012	−0.206	.837	0.042	0.800	.424
	Numeric Sequences	−0.022	−0.365	.715	0.073	1.352	.178
	Expressions	**0.208**	**3.376**	**<.001**	0.097	1.735	.084
	Movements and Shapes	−0.085	−1.409	.160	0.024	0.442	.659
	Analogies	0.021	0.342	.733	−0.036	−0.647	.518
	Calculus	0.159	2.102	.036	**0.362**	**5.524**	**<.001**
	Cubes Sequences	0.081	1.308	.192	0.060	1.071	.285
	Problems	0.109	1.336	.183	0.160	2.152	.032

*Note.* Significant statistics in bold (*p* < .001).

**Table 4:** Means, standard deviations (SDs) in cognitive subtests and school achievement in Portuguese and Mathematics curricular subjects by school grade (from tenth to twelfth school grades).

	Grades		
Subtests	Tenth Grade	Eleventh Grade	Twelfth Grade
Synonyms	10.13 (4.05)	11.29 (3.95)	12.18 (4.00)
Analogies	12.42 (3.74)	12.76 (3.91)	12.71 (3.51)
Expressions	14.70 (4.00)	15.99 (3.68)	16.76 (2.56)
Figures Rotation	10.57 (3.87)	10.70 (3.77)	11.10 (3.91)
Cubes Sequences	10.72 (3.91)	10.84 (3.77)	11.78 (3.84)
Movements and Shapes	10.15 (3.67)	10.52 (3.73)	11.71 (3.70)
Calculus	11.96 (5.25)	12.90 (5.27)	13.12 (5.27)
Numeric Sequences	11.80 (5.48)	12.08 (5.43)	12.16 (5.20)
Problems	9.84 (4.70)	10.79 (5.14)	11.54 (4.68)
**School achievement**			
Portuguese	11.76 (2.80)	12.87 (2.76)	13.58 (2.52)
Mathematics	11.40 (3.69)	12.03 (3.65)	13.54 (3.69)

**Table 5:** Correlation coefficients between cognitive subtests and academic achievement in Portuguese and Mathematics by school grade (from tenth to twelfth grade).

Subtests	Tenth Grade		Eleventh Grade		Twelfth Grade	
	Port	Math	Port	Math	Port	Math
Synonyms	0.45 ***	0.35 ***	0.44 ***	0.33 ***	0.45 ***	0.37 ***
Analogies	0.38 ***	0.29 ***	0.40 ***	0.28 ***	0.31 **	0.29 ***
Expressions	0.34 ***	0.23 ***	0.35 ***	0.19 ***	0.34 ***	0.27 ***
Figures Rotation	0.08 *	0.14 ***	0.12 *	0.13 ***	0.12 **	0.22 ***
Cubes Sequences	0.24 ***	0.29 ***	0.26 ***	0.25 ***	0.28 ***	0.38 ***
Movements and Shapes	0.16 ***	0.19 ***	0.23 ***	0.25 ***	0.20 ***	0.30 ***
Calculus	0.36 ***	0.34 ***	0.35 ***	0.29 ***	0.31 ***	0.31 ***
Numeric Sequences	0.18 ***	0.19 ***	0.22 ***	0.15 ***	0.24 ***	0.20 ***
Problems	0.38 ***	0.35 ***	0.35 ***	0.26 ***	0.30 ***	0.35 ***

* *p* < .05, ** *p* < .01, *** *p* < .001 (two-tailed test). *Note*. Port, Portuguese school achievement; Math, Mathematics school achievement.

**Table 6:** Summary of the regression analysis coefficients by school grade (tenth to twelfth grade) and school curricular subjects.

		Portuguese			Mathematics		
		β	*t*	*p*	β	*t*	*p*
**Tenth grade**	Synonyms	**0.233**	**5.753**	**<.001**	**0.218**	**4.903**	**<.001**
	Figures Rotation	−0.074	−1.888	.059	−0.038	−0.889	.374
	Numeric Sequences	−0.001	−0.030	.976	0.021	0.495	.621
	Expressions	**0.145**	**3.641**	**<.001**	0.005	0.114	.909
	Movements and Shapes	−0.111	−2.580	.010	−0.014	−0.303	.762
	Analogies	0.099	2.360	.019	0.026	0.572	.568
	Calculus	0.129	2.647	.008	0.149	2.815	.005
	Cubes Sequences	0.048	1.084	.279	0.110	2.321	.021
	Problems	0.140	2.755	.006	0.104	1.885	.060
**Eleventh grade**	Synonyms	**0.287**	**7.546**	**<.001**	**0.240**	**5.548**	**<.001**
	Figures Rotation	−0.080	−2.198	.028	−0.054	−1.337	.182
	Numeric Sequences	−0.020	−0.525	.600	−0.060	−1.418	.157
	Expressions	0.090	2.371	.018	−0.061	−1.428	.154
	Movements and Shapes	0.043	1.067	.286	0.144	3.150	.002
	Analogies	**0.164**	**4.115**	**<.001**	0.097	2.163	.031
	Calculus	0.114	2.348	.019	0.169	3.064	.002
	Cubes Sequences	0.019	0.453	.651	0.078	1.702	.089
	Problems	0.059	1.190	.234	−0.025	−0.455	.649
**Twelfth grade**	Synonyms	**0.322**	**6.708**	**<.001**	**0.245**	**4.476**	**<.001**
	Figures Rotation	−0.065	−1.244	.214	−0.029	−0.487	.627
	Numeric Sequences	0.051	1.064	.288	0.005	0.098	.922
	Expressions	**0.161**	**3.396**	**<.001**	0.083	1.552	.121
	Movements and Shapes	−0.080	−1.342	.180	0.094	1.392	.165
	Analogies	0.085	1.674	.095	0.057	1.015	.311
	Calculus	0.101	1.632	.103	0.033	0.491	.624
	Cubes Sequences	0.092	1.674	.095	0.081	1.305	.193
	Problems	0.059	0.897	.370	0.134	1.881	.061

*Note.* Significant statistics in bold (*p* < .001).

## Data Availability

Data shared is in accordance with consent provided by participants on the use of confidential data. The publication of this data does not compromise the anonymity of the participants or breach local data protection laws. Access to data is restricted to protect confidential or proprietary information.
